# Supplementary material for: Chromothripsis during telomere crisis is independent of NHEJ, and consistent with a replicative origin
Source: Genome Res. 2019 May;29(5):737–49. doi: 10.1101/gr.240705.118 (PMC6499312; doi:10.1101/gr.240705.118)
Supplement: Supplemental Material [file supp_gr.240705.118_Supplemental_file_1.zip › contigs/annotated_contigs/DB53/contig.3.DB53_length_834_mean_cov_20.1738609113.docx]

**DB53_length_834_mean_cov_20.1738609113**

```
TCCCCTTTTCTGTCTAGCTCTACTCTCTGTCACTGCATCTGCTCCTCCATCTCCCAGAGACAGCCAGTCTCTGGATTTCTCCTTTTCTC
 >chr8:81613114-81613548 + E=3e-246
CTAGGCAGTTAGAGGCTAGTACTTCTGACCTAGAACAGCTTGCTGCTGGCTACTGCTTCCCTCCCGCCAGGTCCACTGGTGGCTCAGCG

GGGTTCTCCCTTTACCAGCATCCCTGAATCCCTGGCCTTGCTAGCCTTCTGCTCCACTGGCCCAGAAGAACCAAACCCTGGCTGAATCC

TCCTGTGCTGCTCTTTACTGTCCACCTCAGGGCAGTCAGTGCTGATGGGAAACAGCGAATGACTCTGCTGGCCGGGCCCATCCTCGGCA

CGCATCCTACCTGCACTTGGCCTCAGCAGGCCCTTCACCCGCCCTCGCTCACCACAACTGCCCGCTTCTAGCCAGGAT|CC|TTCCCCC
                                                                                 >chr8:
ATGTATGTTCTTGGAGGCTTGTTGAAGATCAGTTTCCTGTAGATGTGTGAACTTATGTCTGGGCTCTCTCTTCTGTCACATTGGTCTGT
77853915-77854202 + E=9e-158
TTGTGTGTTTCTATGCCAGTACCATGCTGTTTAAGTTAACATAAGCTCTGTAGTGTATTTTGAGGTCAGGTAATGTAATGCCTCCAGAT

TTTGTTTGTTTGTTTGTTTACTTAGGATTTCTTTGGCTATTCGGGGTCTTTTGTGGTGTCATACAAATTTTAGGATTGTTTTCCTATTT

CTGTGAAGAATGC|T|ACCCAGGCTGCGGTGCGGTGGCGCTCTCTCGGCTCACTGAAACCTCTGCTTCCCGGGTTCCAGTGATTCTTCT
               >chrUn_gl000220:130249-130359 + E=3e-39
TTGGTAGCTGGGAGTCCAGGTGCGGACCATGCCGGCC
```
